# Estimation of Individual Neonatal Survival Using Birthweight and Gestational Age: A Way to Improve Neonatal Care

**Published:** 2008-03

**Authors:** Francisco Mardones, Guillermo Marshall, Paola Viviani, Luis Villarroel, Barton R. Burkhalter, José-Luis Tapia, Jaime Cerda, Trinidad García-Huidobro, Constanza Ralph, Enrique Oyarzín, Francisco Mardones-Restat

**Affiliations:** 1 Department of Public Health, Faculty of Medicine; 2 Faculty of Mathematics, Pontificia Universidad Católica de Chile, Santiago, Chile; 3 Quality Assurance Project, University Research Co, Bethesda, Maryland, USA; 4 Departments of Pediatrics; 5 Obstetrics and Gynecology, Faculty of Medicine, Pontificia Universidad Católica de Chile, Santiago, Chile

**Keywords:** Birthweight, Gestational age, Neonatal mortality, Chile

## Abstract

The study was conducted to determine the combined effect of birthweight and gestational age at birth on neonatal mortality using individually-identified livebirths. Logistic regression was used for studying the interactive effect of birthweight and gestational age on the individual probability of neonatal death. All livebirths from Chile in 2000 were included in a linked file. Odds ratio models for birthweight and gestational age were developed for each sex. The probability of neonatal death by sex was presented using contour plots. The models were statistically significant, and odds ratios were different and non-linear for the effects of birthweight and gestational age. Contour plots of constant neonatal mortality according to birthweight and gestational age were presented; they were similar for each sex. A single graph for both sexes that estimates the survival potential of infants born too early or too small would improve neonatal care in developing countries.

## INTRODUCTION

Birthweight and gestational age at delivery are major determinants of survival, morbidity, and nutritional status during the neonatal and postneonatal periods in both developed and developing countries (1-3). Over three decades ago, Lubchenko *et al*. used neonatal mortality data by birthweight and gestational age to evaluate the quality of perinatal care in a US hospital (4). In the following decade, those and subsequent findings in different countries were considered to be the standards by which birthweight and gestational age are related to neonatal survival and judging the adequacy of neonatal care (4-9). However, worldwide advances in neonatal intensive care have clearly declined a newborn's risk of death during the neonatal period and, therefore, made those early results obsolete. For example, most improvement in neonatal survival in the USA since the early 1980s seems to be related to decreasing birthweight-specific mortality rates among very low-birthweight (LBW), preterm, and LBW infants, which occurred during a period of increasing preterm and LBW rates (10).

Neonatal mortality in Chile dropped dramatically from 37.8 in 1960 to 4.9 in 2003 (11). The initial decrease from 1960 to the 1980s has been mainly attributed to a lower incidence of LBW from over 10% to around 6% (12). Further reductions in national incidence of LBW were not observed during 1990–2003, with stable figures hovering around 5.6%, while the preterm incidence increased slightly from 5.6% to 6.5%. Nevertheless, during 1990–2003, rates of neonatal and early neonatal mortality declined from 8.5 to 4.9 and from 6.8 to 3.8 respectively (11). Those declines were not associated with decreases in the proportion of LBW and preterm infants but rather with declines in birthweight-speci-fic and gestational age-specific mortality rates (13). It is possible that the huge reduction in neonatal mortality since 1960, together with stable figures for LBW and a slight increase in preterm incidence in the last 15 years may still not be well-acknowledged in Chile. This possibility has recently been studied for the US because it may affect appropriate neonatal practice due to the underestimation of the survival potential of an infant born too early or too small (14). This underestimation has been associated with a decreased use of various appropriated interventions (15). Therefore, recent national estimates of early survival according to birthweight and gestational age are needed for Chile and other countries; developing countries may have more room for improvement in neonatal survival than developed countries.

This report presents Chilean neonatal morta-lity data from the 2000 period linked file. In this linked file, information from the death certificate is matched to information from the birth certificate for each infant who died during the neonatal period, which enables the calculation of mortality risks as a function of additional variables available from the birth certificate. The interactive effect of birthweight and gestational age on the individual probability of neonatal death was calculated for each sex. The resulting models are presented as odds ratios (ORs) and contour plots.

## MATERIALS AND METHODS

The interactive effect of birthweight and gestational age on the probability of neonatal death was assessed using logistic regression. The study of the interactive effect of sex was not included in the logistic regression, and results were presented separately by sex.

The study obtained data on all livebirths (248,893) and their corresponding neonatal deaths (1,463) as registered for 2000 in the Chilean Civil Registry Service. Therefore, survivors were 247,430 cases. Survivors were those born during 2000 but not dying during the neonatal period. Meanwhile, neonatal deaths—defined as those dying less than 28 days after delivery—included cases who were born and died during 2000 and also included 11 cases who died in January 2001. Survivors and neonatal deaths were calculated using the individual identification number recorded in the birth and death certificates. This unique number has been recorded in Chile since the 1990s, and its presence enabled us to link the livebirth and neonatal death files.

Birthweight is determined at maternity hospitals immediately after delivery by trained personnel, using beam scales and standard procedures. Gestational age is estimated by the date of the last menstrual period, and for uncertain dates, an early ultrasound test allows for corrections. Ultrasound is available for all pregnant women before 20 weeks of gestation in Chile; when the latter is not performed due to a late pregnancy check-up, a postnatal clinical examination of the newborn done by the physician-in-charge in the maternity hospital is used for estimating gestational age at birth. Only a few women in Chile do not have pregnancy check-ups before 20 weeks of gestation.

Non-registered livebirths and neonatal deaths are assumed to be negligible. About 30 years ago, the Inter-American Study of Childhood Morta-lity found high rates of under-registration of neonatal deaths in Chile (16). Partially as a result of this finding, Chile instituted various policies and practices that all but eliminated non-registration of livebirths and neonatal deaths. In 2000, 99.8% of all livebirths had deliveries attended by professionals, and 99.0% took place in maternity hospitals (17). All public and private hospitals in Chile are required to file a delivery certificate. Death certificates endorsed by medical doctors are also legally required. Both delivery and death certificates must be registered with the Civil Registry Service, generally located within maternity hospitals, thus facilitating immediate registration of births and deaths. In addition, recording of births is encouraged by the monetary incentives of the social security system, and registration of death is required before internment of the body. Chile has been reported as having the lowest under-registration rate for all deaths in Latin America (18). Thus, the number of unregistered births and deaths is likely to be very small or non-existent.

We studied the relationship of birthweight, gestational age at delivery, and sex within neonatal mortality, as explained below. Only livebirths and neonatal deaths with complete information on these three items were included in the study. Our analyses included all neonatal deaths and a 5% random sample of the survivors.

Our purpose was to develop a model that reliably predicts the risk of neonatal death as a function of birthweight and gestational age for males and for females. Multivariate generalized additive models were developed to ascertain the possible influence of these variables on neonatal death risk for each sex (19). Each model estimates the logit of neonatal survival or death, log_e_ {p/(1-p)}, where p is the probability that a newborn will die during the neonatal period. OR is the ratio between the odds in favour of exposure to risky birthweight or risky gestational age among neonatal deaths and the odds in favour of exposure to risky birthweight or risky gestational age among survivors. These models were built using the smoothing function LOESS (locally-weighted least squares regression) (19).

The resulting two models by sex were presented using contour plots for the probability of neonatal death; a single graph that includes both sexes is also presented. These contour lines reflect constant neonatal mortality rates over different combinations of birthweight and gestational age. Logistic models and contour plots were developed using the S-PLUS software (20).

## RESULTS

Combined information on birthweight, gestational age at delivery, and sex was available for 246,975 (99.82%) of 247,430 survivors and 1,425 (97.40%) of 1,463 neonatal deaths. All 455 survivors not fulfilling this requirement had information on sex and among them, 244 survivors had information on birthweight with a mean value of 3,293±595 g, and only 10 cases had information on gestational age with a mean value of 34.8±4.08 weeks. In 38 neonatal deaths not fulfilling the requirement of combined information for birthweight, gestational age at delivery, and sex, information on sex was present in all of them. Just 19 cases had information on birthweight with a mean value of 1,888±1,070 g, and 17 cases had information on gestational age with a mean value of 33.9±5.2 weeks.

The study population included only cases with complete information on the three relevant variables. Table 1 shows that the mean values of gestational age and birthweight were lower for neonatal deaths; the proportions of females were also lower for neonatal deaths. Table 2 includes the number of livebirths, the number of neonatal deaths, and the neonatal mortality rates, according to the cate-gories of birthweight and gestational age. Most (85.02%) livebirths were in the category of 2,500–3,999 g, while 29.75% of neonatal deaths fell in this category. The minority (0.89%) of livebirths was in the category of <1,500 g, while 46.60% of neonatal deaths fell in this category.

**Table 1:** General description of survivors and neonatal deaths with complete information on birthweight, gestational age, and sex, Chile, 2000

Livebirth information	Survivors (n=246,975)	Neonatal deaths (n=1,425)
Gestational age (w)	38.80±1.61	31.96±6.06
Birthweight (g)	3,357.84±528.86	1,846.0±1095.88
Females (%)	120,261 (48.69)	637 (44.70)

Values for the first two variables are mean±SD; Values for females are no. and %

**Table 2:** Neonatal mortality according to birthweight and gestational age categories, Chile, 2000

GA (weeks)		Birthweight (g)						Total
		<500	500–999	1,000–1,499	1,500–2,499	2,500–3,999	4,000+	
<22	LB ND NMR	24 24 1,000.00	41 39 951.22	2 1 500.00	1 0 0.00	1 0 0.00	0 0 -	69 64 927.54
23–25	LB ND NMR	8 8 1,000.00	302 248 821.19	6 1 166.67	2 1 500.00	5 1 200.00	0 0 -	323 259 801.86
26–28	LB ND NMR	3 1 333.33	311 112 360.13	313 61 194.89	11 3 272.73	6 0 0.00	0 0 -	644 177 274.84
29–31	LB ND NMR	4 3 750.00	118 37 313.56	610 60 98.36	477 40 83.86	42 9 214.29	1 0 0.00	1,252 149 119.01
32–34	LB ND NMR	0 0 -	19 5 263.16	340 41 120.59	2,845 107 37.61	466 11 23.61	4 0 0.00	3,674 164 44.64
35–37	LB ND NMR	0 0 -	5 2 400.00	84 18 214.29	5,056 112 22.15	20,437 95 4.65	501 5 9.98	26,083 232 8.89
38–42	LB ND NMR	3 0 0.00	9 0 0.00	11 3 272.73	2,508 48 19.14	190,198 308 1.62	23,586 21 0.89	216,315 380 1.76
43 +	LB ND NMR	0 0 -	0 0 -	0 0 -	0 0 -	31 0 0.00	9 0 0.00	40 0 0.00
Total	LB ND NMR	42 36 857.14	805 443 550.31	1,366 185 135.43	10,900 311 28.53	211,186 424 2.01	24,101 26 1.08	248,400 1,425 5.74

GA=Gestational age (weeks), LB=Livebirths, ND=Neonatal deaths, NMR=Neonatal mortality rate (1:1,000 LB)

**Fig. 1:**
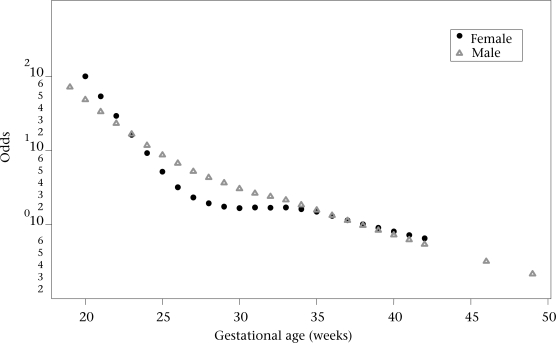
Odd ratios (log ORs) for gestational age in the multivariate model

**Fig. 2:**
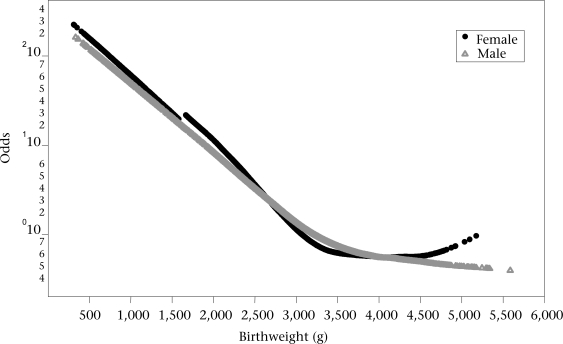
Odd ratios (log ORs) for birthweight in the multivariate model

Results of logit multivariate analyses using LOESS demonstrated that the associations of birthweight and gestational age were non-linear when each variable was adjusted by the effect of the other. The ORs of the two final multivariate models are presented as functions of birthweight and gestational age in Figure 1 and 2, which depict information for each sex. ORs were quite similar for the two sexes.

**Fig. 3:**
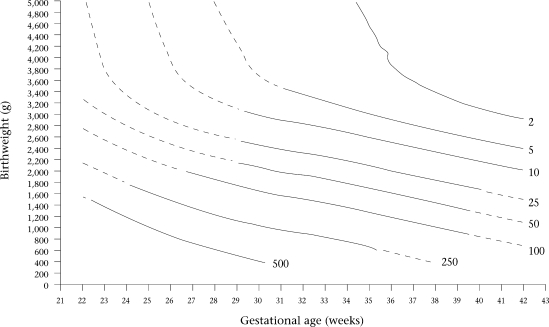
Contour plots of constant neonatal mortality per 1,000 livebirths according to birthweight and gestational age for males

**Fig. 4:**
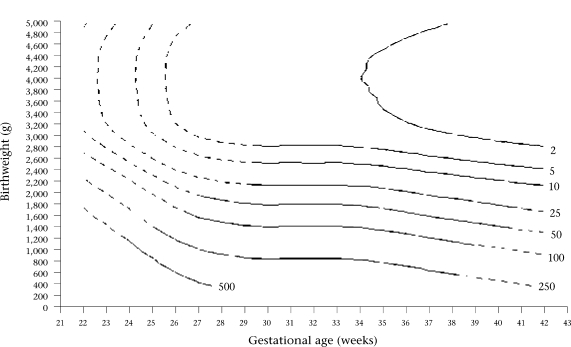
Contour plots of constant neonatal mortality per 1,000 livebirths according to birthweight and gestational age for females

**Fig. 5:**
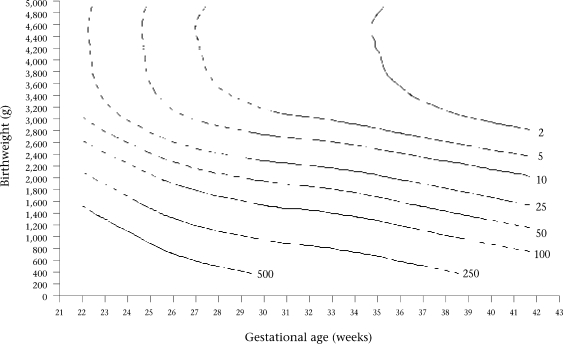
Contour plots of constant neonatal mortality per 1,000 livebirths according to birthweight and gestational age for females

Contour plots of constant neonatal mortality according to birthweight and gestational age are presented for each sex in Figure 3 and 4. The area over each contour plot of neonatal mortality rate meant that the specific neonatal mortality, i.e. 2, 5, 10, etc., per 1,000 livebirths, corresponded to that mortality risk for the specific birthweight and gestational age values included in that space of the graph. Contour plots of constant neonatal mortality according to birthweight and gestational age were very similar between both sexes, with the exception of very low mortality rates. Therefore, contour plots are presented for both sexes combined in Figure 5.

Continuous and discontinuous lines are presented for the contour plots drawn by the models (Fig. 3, 4, and 5). The estimates presented with continuous lines belong to the models developed with actual information from cases of this study. The estimates presented with discontinuous lines belong to the extrapolation of the models.

## DISCUSSION

The completeness of Chilean birth and death records enabled us to obtain data on neonatal deaths, birthweight, gestational age, and sex for nearly all livebirths in Chile in 2000. Using logit regression analysis of these data, we obtained formulae of the probability of neonatal death as a function of birthweight and gestational age for males and females. Thus, the survival probability can be calculated if the birthweight, gestational age, and sex are known. We have used these formulae for creating graphs with contour lines of constant neonatal death probability in all possible combinations of birthweight and gestational age that clinicians can conveniently use.

Mathematical models permit the general tendency of the associations between the independent variables and neonatal mortality to be ascertained. The discontinuous lines in the figures are presumed to be tentative. Although estimates in areas of birthweight for gestational age with a low number of cases may also be tentative, they are the best possible estimation available that could be used by clinicians. It seems better to have an estimation derived from the actual data rather than not having it, considering that single clinical judgments have been shown, on average, to estimate lower survival probabilities than the data show (14,15). Furthermore, Figure 1 and 2 show that OR values for ranges of extremely low or extremely high birthweight and gestational age follow the general trend of the data for males and females in the sample studied. The small kinks that are present in the contour plots for neonatal mortality level 2 in Figure 3, 4, and 5 reflect certain discontinuities in the data. It can be appreciated that there is a clear general pattern despite the discontinuities.

These results can help improve the quality of newborn care, especially for prematures. Clinicians who correctly estimate neonatal survival probabilities of premature newborns tend to provide more appropriate care than those who underestimate survival probability. It has been observed that neonatologists with the correct estimation of neonatal survival intervene more often with appropriate invasive therapies, such as mechanical ventilation, cardiopulmonary resuscitation, inotropes, and intravenous fluids, than clinicians who are not well-informed about survival probabilities (14,15).

This Chilean data showed birthweight and gestational age distributions of livebirths and neonatal deaths similar to Hispanics in the USA (10). Both datasets showed a similar pattern of higher neonatal mortality associated with lower birthweight and gestational age, and in both, the proportion of female survivors was lower than 50%, a usual observation due to the higher proportion of male newborns, which, in turn, have higher neonatal mortality than females (21). Nevertheless, neonatal mortality rates for the birthweight-gestational age subcategories in the US Hispanics are generally lower than in the Chilean data, probably reflecting on average better medical technology in the USA.

Developing countries lacking complete information on birthweight and gestational age for assessing their own estimates may benefit from this proposal prepared with Chilean data.

The US study analyzed its data by subcategories of birthweight and gestational age, resulting in a tabular presentation of neonatal mortality rates for each birthweight-gestational age subcategory. We believe our approach using logit regression is statistically superior to the US study approach, because it statistically smoothes neonatal mortality over the entire range of birthweight and gestational age. It can, therefore, calculate the probability of a neonatal death for a particular newborn from the birthweight and gestational age of that individual rather than estimating the probability of neonatal death only for birthweight-gestational age subcategories.

The distribution of neonatal mortality by birthweight and gestational age, as reflected by the contour plots, is very similar for both sexes, except for small but significant differences for low neonatal mortality (between 2 and 5) when birthweight and gestational age are large. Therefore, we have combined the results for both sexes into one contour graph (Fig. 5) for convenient use by clinicians, because the quality of clinical treatment has been shown to be better for cases with low birthweight and gestational age if survival probability is estimated accurately (14,15). Thus, the differences in survival probability between males and females for large birthweight and gestational age that are masked in the combined contour graph are not clinically important.

In addition to increasing the use of appropriate interventions, this predictive instrument can also serve to evaluate the quality of clinical care by estimating the probability of neonatal death according to birthweight and gestational age in any maternity hospital (4). By not including foetal deaths, there is probably a bias against tertiary-care hospitals vs delivery at home or at primary-care hospitals. Some very premature or very low-birthweight foetal deaths being delivered in a primary-care hospital may be born alive if they happen to be delivered in a tertiary-care hospital. This is the case because obstetric care is better there. They may also be more likely to survive at least 28 days after birth due to better neonatal care. However, the combination of these two possible effects on neonatal mortality rates in tertiary-care hospitals and in primary hospitals or care at home is difficult to estimate. There is a small chance that a tertiary-care hospital could have better obstetric care but similar or even worse neonatal care than primary hospitals or care at home, or the inverse result, but we do not believe either assumption is justified without sound evidence. Therefore, we suggest that foetal and neonatal mortality tendencies be compared at the local and the national level whenever possible. Inclusion of foetal deaths in this study would have made this instrument not useful for many countries where a substantial number of stillbirths go unreported, as has been well-documented for many countries worldwide (22); this is an important reason for not including perinatal deaths in the dependent variable of this study.

Of the two factors, birthweight is the most important influence on risk of neonatal mortality for term and preterm infants when birthweight is less than about 3,000 g, but gestational age becomes of more importance as birthweight exceeds 3,000 g. In Figure 5, the vertical contour lines when birthweight is over 3,000 g signify that higher birthweight for a particular gestational age does not increase survival probability but increased gestational age does. This finding supports the view that clinicians should not induce delivery just based on estimations of birthweight.

There are several potential sources of bias in this study, including unregistered livebirths, missing data, measurement errors in birthweight and gestational, and sampling error.

Registration of livebirths appears to be universal, and it is widely accepted that Chile is now in this position because of financial incentives to register livebirths and the need for a legal burial. Of the registered livebirths, 0.18% of survivors and 2.6% of neonatal deaths lacked registered information on birthweight, gestational age, or sex and were excluded from the study (Table 1). These added 493 livebirths (455 survivors plus 38 neonatal deaths) reached a similar figure to the group of non-professionally-attended livebirths explained in the Methods section (0.2% of all Chilean livebirths). It could be speculated that they belong to the same group and could not have complete information on birthweight, gestational age, and sex due to this situation. Survivors with missing data were a very small proportion, and of them, 244 cases had information on birthweight alone with very similar mean±SD to the total population of survivors, not showing a special bias in this subgroup.

Neonatal deaths with missing registered information on the above-mentioned three variables were 2.6% of all cases, a much higher proportion. Neonatal mortality per 1,000 livebirths calculated with these 38 cases, using the 455 survivors as denominator of the rate, reached a much higher rate (83.5) than that for the entire year 2000 figure (5.74), and this study may, therefore, slightly overestimate neonatal survival probabilities. This fact is, nevertheless, not concentrated in neonatal deaths with different birthweight or gestational from the total group of neonatal deaths in 2006. This can be estimated from the 17 and 19 cases with information on birthweight or gestational age alone that had very similar means±SD than the total population of neonatal deaths.

Regarding possible measurement errors of birthweight and gestational age, Chile uses standard procedures for determining birthweight and gestational age as described above, and there is no reason to believe that possible systematic errors may bias the results. Gestational age, usually estimated by the date of the last menstrual period, as the benchmark method of measurement and as the single method of measurement has been considered to be misleading (23,24). However, in many countries around the world, including Chile, gestational age is presently estimated by the date of the last menstrual period but confirmed by an early transvaginal ultrasound test.

From a biological standpoint, the duration of pregnancy is the elapsed time between conception and delivery of the infant. This definition has a limited clinical application because of the difficulties in accurately establishing when fertilization occurred. Because the ovulation date and the date of fecundating coitus are rarely known, menstrual age is the standard method used for expressing the duration of pregnancy. All published charts relating sonographic findings to gestational age are, in fact, based on the menstrual age. To understand the accuracy and limitations of ultrasound dating, it is important to know how tables for dating are constructed. Sonographic measurements of patients with accurate dating are performed to establish the relationship between age and the size of foetal biometric parameters. This relationship is established through regression analysis. The crown-rump measurement in the first trimester of pregnancy is predictive of menstrual age with an error of three days (90% confidence limits) from the seventh week to the 10^th^ week. (25). After 11 weeks and before 20 weeks, the biparietal diameter and the femur length are the best parameters in predicting gestational age (26,27). All women are expected to have at least three ultrasound tests: the first at 11–14 weeks of gestation, the second at 18–24 weeks of gestation, and the third at 30–34 weeks of gestation. Although this norm is not compulsory, an early ultrasound test is, in fact, a medical indication in the case of uncertain dates. Therefore, important errors in measurements of gestational age are very unlikely in this study.

Random sampling of 5% of the survivors permitted the estimation of neonatal death probabilities with a sample error lower than 1% (28); this fact supports sample representativity.
